# Automated radiomics pipeline integrating TotalSpineSeg for Pfirrmann grading of lumbar disc degeneration with SHAP-based interpretability

**DOI:** 10.3389/fmed.2026.1929252

**Published:** 2026-09-29

**Authors:** Dongqi Guo, Yuan Wang, Chen Yang, Jiawen Zhan, Xue Han, Yi Wang, Zhaoshu Huang, Ping Zhang

**Affiliations:** 1 Department of Pathology, Wangjing Hospital, China Academy of Chinese Medical Sciences, Beijing, China; 2 Tianjin University of Traditional Chinese Medicine, Tianjin, China; 3 Institute of Chinese Materia Medica, China Academy of Chinese Medical Sciences, Beijing, China; 4 Institute of Orthopaedics and Traumatology, China Academy of Chinese Medical Sciences, Beijing, China; 5 Department of Sports Medicine, Wangjing Hospital, China Academy of Chinese Medical Sciences, Beijing, China; 6 Department of Radiology, Wangjing Hospital, China Academy of Chinese Medical Sciences, Beijing, China; 7 Department of Radiology, The Affiliated Hospital of Guizhou Medical University, Guiyang, Guizhou, China

**Keywords:** automated segmentation, explainable artificial intelligence, lumbar disc degeneration, machine learning, Pfirrmann grading, radiomics, SHAP analysis, T2-weighted MRI

## Abstract

**Background:**

Pfirrmann grading of lumbar disc degeneration (LDD) is constrained by inter-observer variability. Although radiomics enables objective quantification, the manual segmentation required by existing models is time-consuming and undermines feature reproducibility. TotalSpineSeg, an nnU-Net-based tool, allows fully automated segmentation, yet its integration with radiomics for Pfirrmann grading remains unexplored. This study aimed to develop an automated radiomics pipeline incorporating TotalSpineSeg for Pfirrmann grading of LDD, and to assess whether three-dimensional features contributed discriminative information beyond mid-sagittal two-dimensional radiomics, simple measurements, and frozen ResNet50 embeddings (ImageNet and RadImageNet). Interpretability analysis was applied to identify candidate imaging biomarkers.

**Methods:**

Magnetic resonance imaging (MRI) data from 210 patients with LDD yielded 630 disc samples (L3–S1, Pfirrmann Grades I–V). Segmentation was performed automatically with TotalSpineSeg. Of 1,774 extracted radiomics features, 517 were retained through a multi-step selection process. Ten machine learning algorithms were evaluated using a patient-level 80:20 train–test split with 5-fold cross-validation, and SHapley Additive exPlanations (SHAP) analysis was applied for feature interpretation.

**Results:**

Automated segmentation averaged 6.9 s per case. The CatBoost model achieved a macro-averaged area under the curve (AUC) of 0.936 (95% confidence interval [CI]: 0.913–0.958) for three-dimensional radiomics, followed by mid-sagittal two-dimensional radiomics (0.916), frozen convolutional neural network (CNN) embeddings (RadImageNet, 0.874; ImageNet, 0.864), and the strongest simple measurement (0.760). For the three-dimensional model, sensitivity and specificity were 63.9% and 93.1%, grade-specific AUCs ranged from 0.876 to 0.978, and SHAP analysis identified the Laplacian of Gaussian first-order minimum, Flatness, and gray-level co-occurrence matrix (GLCM) Imc2 as candidate biomarkers.

**Conclusions:**

Integrating TotalSpineSeg with radiomics offers an efficient and interpretable approach to Pfirrmann grading. Three-dimensional features contributed the greatest discriminative information, with a modest increment over two-dimensional radiomics and a significant advantage over simple measurements and frozen ResNet50 embeddings. The candidate biomarkers identified are pathophysiologically meaningful features for LDD assessment.

## 1 Introduction

Lumbar disc degeneration (LDD) and its secondary conditions are increasing in prevalence and produce a spectrum of symptoms ranging from pain and numbness to motor weakness and paralysis, which severely impair patients' quality of life. According to the Global Burden of Disease Study, approximately 619 million people worldwide were affected by low back pain in 2020, a figure projected to reach 843 million by 2050, making it one of the foremost contributors to the global disability burden (1). Early and accurate evaluation of LDD severity is therefore critical to guiding clinical decision-making.

The Pfirrmann classification system is widely adopted as the standard magnetic resonance imaging (MRI)-based method for grading LDD severity. Its pathophysiological rationale is that the intervertebral disc undergoes progressive biochemical and structural changes as degeneration advances, including decreased water content, proteoglycan degradation, and collagen disorganization, which manifest as signal-intensity and morphological abnormalities on T2-weighted MRI (2, 3). The system stratifies disc degeneration into five grades on the basis of signal intensity, structural appearance, nucleus-annulus differentiation, and disc height (4). Despite its broad clinical utility, this visual approach is inherently subjective: reported inter-observer weighted kappa values range from 0.65 to 0.83 (5, 6), with disagreement occurring most often between adjacent grades, where imaging characteristics overlap substantially. Moreover, the qualitative nature of Pfirrmann grading precludes continuous quantification, limiting its sensitivity for detecting early degenerative changes and monitoring disease progression (7). An additional limitation is that the criteria are applied only to the mid-sagittal T2-weighted image, potentially omitting through-plane intensity and shape information (4, 8, 9). The modified Pfirrmann system provides finer stratification for elderly patients but does not resolve these limitations (5, 10). Together, these constraints underscore the pressing need for objective, reproducible, and quantitative methods to standardize LDD assessment.

Simple measurements such as disc-height indices (11) and peak signal-intensity features (7) yield objective scores but predominantly capture a single axis of degeneration. Mid-sagittal radiomics combines multiple descriptors (8) yet remains confined to the grading plane. End-to-end convolutional neural network (CNN) classifiers achieve high reported accuracy (12–16) but rarely yield inspectable intensity, shape, and texture quantities.

Three-dimensional radiomics extracts quantitative features from the entire disc volume rather than the mid-sagittal plane alone, capturing intensity distributions, morphological attributes, and textural patterns that frequently elude visual inspection (17, 18). Unlike conventional visual assessment, it yields objective, quantitative measurements that are inherently more reproducible (19). Prior radiomics studies of intervertebral disc assessment have shown that machine learning classifiers can achieve area under the curve (AUC) values of 0.85 to 0.92 for degeneration grading (12, 20). Clinical translation, however, has been substantially hampered by a critical methodological bottleneck: the need for manual region-of-interest (ROI) segmentation.

Manual disc segmentation is time-consuming, typically requiring 5–15 min per disc, which has partly contributed to the relatively small sample sizes of existing LDD radiomics studies and, in turn, limited their statistical power and model robustness. It also introduces operator-dependent variability that compromises feature reproducibility (21, 22). Manual delineation has been shown to introduce considerable inter-observer bias, with many radiomic features showing poor robustness to segmentation variation (23); this is especially problematic for texture features, which are highly sensitive to boundary definition (24). The absence of standardized segmentation protocols across institutions further limits the generalizability and external validation of radiomics models (25). Semi-automated approaches reduce processing time but still require manual correction and do not eliminate operator dependency (26).

Deep-learning-based automatic segmentation tools have emerged to address this critical bottleneck. TotalSpineSeg, which combines cascaded nnU-Net models with iterative labeling algorithms, achieves fully automated spine segmentation, with a Dice similarity coefficient of 0.80 for intervertebral discs and 99% labeling accuracy across diverse MRI contrasts and resolutions (27–29). By removing operator dependency, it enables rapid, consistent, and large-scale radiomics analysis. To date, however, TotalSpineSeg has not been applied to radiomics-based LDD classification. The present study therefore aimed to establish an automated radiomics workflow incorporating TotalSpineSeg for multi-class Pfirrmann grade classification. Three-dimensional radiomic features derived from these masks were evaluated against mid-sagittal two-dimensional radiomics, simple measurements, and frozen ResNet50 embeddings obtained from the same masks, to assess whether they contributed discriminative information beyond these alternatives. Furthermore, SHapley Additive exPlanations (SHAP) analysis, grade-wise association tests, comparison against simple measurements, and ablation of a reduced feature subset were performed to enhance model interpretability and identify candidate imaging biomarkers associated with the degree of LDD.

## 2 Materials and methods

### 2.1 Study design

This retrospective study was performed at a single institution and received ethical approval from the Ethics Committee of Wangjing Hospital, China Academy of Chinese Medical Sciences (approval number: WJEC-KT-2024-018-P003). Owing to the retrospective design, the requirement for written informed consent was waived, and all patient data were anonymized prior to analysis. The study was conducted and reported in accordance with the CheckList for EvaluAtion of Radiomics research (CLEAR) guidelines (30; Supplementary Table S1). The technical pipeline is shown in Figure 1.

**Figure 1:**
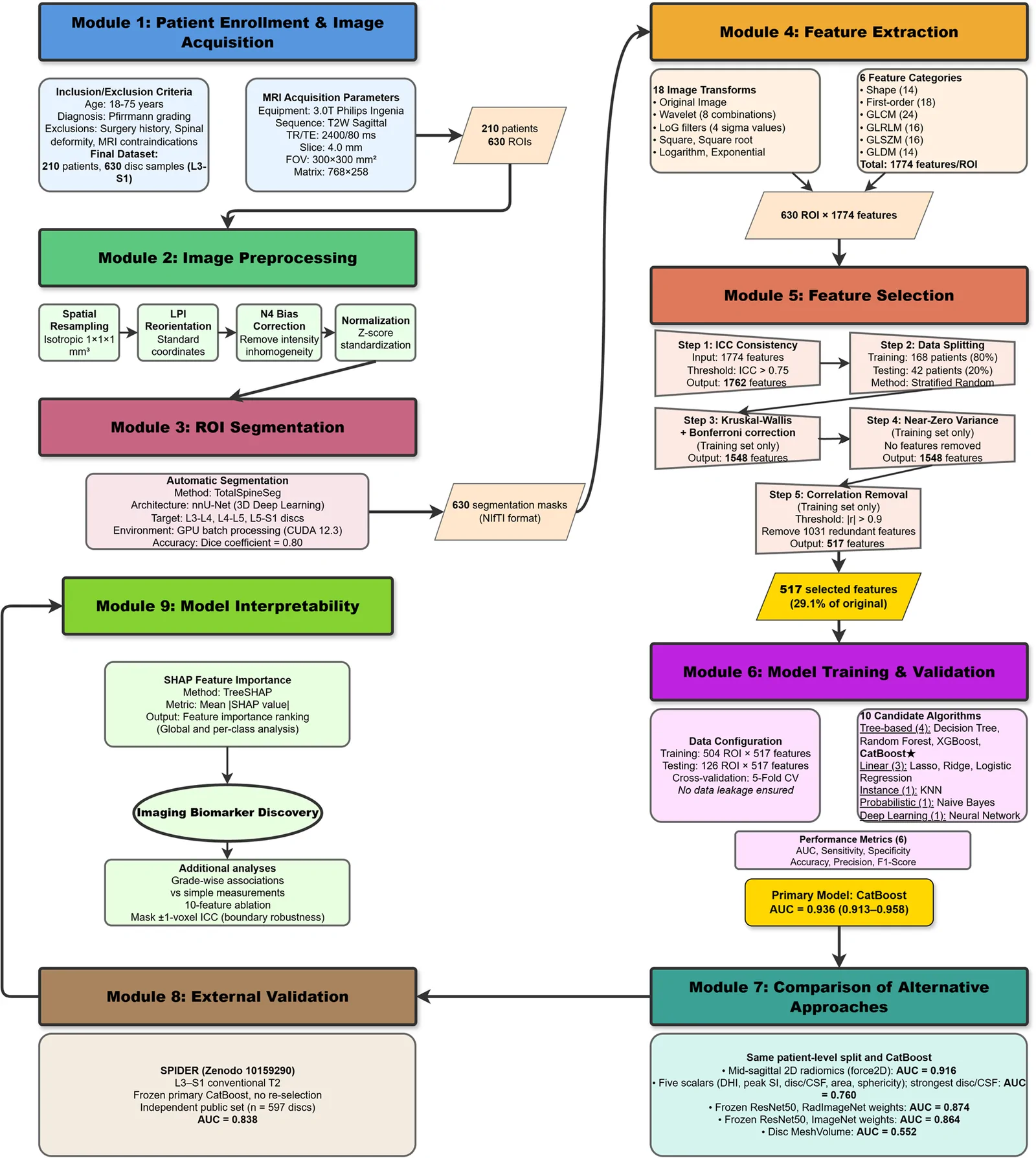
Workflow of this study. ROI, region of interest; ICC, intraclass correlation coefficient; DHI, disc-height index; SI, signal intensity; CSF, cerebrospinal fluid; CNN, convolutional neural network; SHAP, SHapley Additive exPlanations; AUC, area under the receiver operating characteristic curve.

### 2.2 Patient enrollment and image acquisition

Patients who underwent lumbar spine MRI between January 2022 and December 2024 were retrospectively identified from the Department of Sports Medicine, Wangjing Hospital, China Academy of Chinese Medical Sciences. The inclusion criteria were: (1) age 18 to 75 years, regardless of sex; (2) lumbar MRI performed for low back pain or lumbosacral radicular pain; (3) a complete sagittal T2-weighted imaging sequence; (4) adequate image quality, free of significant motion or metallic artifacts and other interfering factors; (5) complete clinical data, including age, sex, symptom duration, and visual analogue scale (VAS) pain score; and (6) clearly identifiable disc structures from L3–L4 to L5–S1 suitable for Pfirrmann grading. The exclusion criteria were: (1) a history of spinal surgery (discectomy, spinal fusion, or fixation) or spinal trauma; (2) non-degenerative spinal pathologies, such as spinal tumors, infections (e.g., pyogenic discitis, tuberculosis), or inflammatory diseases (e.g., ankylosing spondylitis); (3) severe spinal deformities (e.g., scoliosis with a Cobb angle > 20° or spondylolisthesis of grade II or above); and (4) severe systemic diseases (e.g., malignancy, autoimmune disorders) that may affect intervertebral disc metabolism. The final cohort comprised 210 patients, yielding 630 intervertebral disc samples (L3–L4, L4–L5, and L5–S1 levels).

All MRI examinations were performed on a 3.0 T Philips Ingenia scanner (Philips Healthcare, Best, The Netherlands). Sagittal T2-weighted images were acquired with the following parameters: repetition time (TR), 2,400 ms; echo time (TE), 80 ms; slice thickness, 4.0 mm; field of view (FOV), 300  ×  300 mm^2^; and acquisition matrix, 768 × 258.

LDD was assessed using the Pfirrmann classification system (Grades I–V), which evaluates signal intensity, disc structure, nucleus-annulus differentiation, and disc height on T2-weighted images (4). Each disc was graded independently by two radiologists with 8 and 11 years of musculoskeletal imaging experience, respectively. Disagreements were resolved by a senior radiologist with more than 20 years of experience, who independently reviewed the disc to assign the definitive grade. All three observers were blinded to clinical information and to one another's assessments. Inter-observer agreement was assessed using the weighted Cohen's kappa coefficient (*κ*) to account for the ordinal nature of the Pfirrmann system (31); calculations were performed with the psych package (version 2.5.6) in R.

### 2.3 Sample size justification

Sample size adequacy was evaluated against established criteria for radiomics studies. The smallest grade category (Grade V) comprised 27 samples, exceeding the recommended minimum of 20–25 per class (32). Following the patient-level split, the independent test set contained 6, 35, 34, 45, and 6 discs of Grades I–V, respectively. Given the high ratio of candidate features to samples, model development followed the quality principles of the Radiomics Quality Score (33) and prioritized overfitting control through stratified sampling, 5-fold cross-validation, and regularized ensemble algorithms (34).

### 2.4 Image preprocessing

Original MRI images in DICOM format were converted to NIfTI format using dcm2niix as implemented in MRIcroGL (version 1.2.20220720). All images then underwent a series of preprocessing steps before feature extraction. First, images were spatially resampled to an isotropic voxel spacing of 1 × 1 × 1 mm^3^ by B-spline interpolation to ensure consistent spatial resolution for automated segmentation (27, 29). Second, all volumes were reoriented to the Left-Posterior-Inferior (LPI) coordinate system using the nibabel package (version 5.3.2) in Python, standardizing anatomical alignment for subsequent processing. Third, N4 bias field correction was applied with SimpleITK (version 2.5.2) to remove the intensity inhomogeneity caused by magnetic field non-uniformity, a common artifact in MRI (35). Fourth, intensity was normalized by Z-score standardization with a normalization scale of 200 to ensure consistent signal intensities across subjects.

For feature extraction, images were resampled to a voxel spacing of 3 × 3 × 3 mm^3^ using B-spline interpolation (sitkBSpline) (36, 37). Gray-level discretization used a fixed bin width of 5 (38), and a padding distance of 10 voxels was applied to accommodate large sigma values in Laplacian of Gaussian (LoG) filtering.

### 2.5 ROI segmentation

Intervertebral disc segmentation was performed with TotalSpineSeg (version r20250224), an algorithm built on the nnU-Net architecture (version 2.6.2) (27, 29). Trained for comprehensive spine segmentation, it automatically delineates intervertebral discs on MRI. For each patient, segmentation masks were generated for the three target discs (L3–L4, L4–L5, and L5–S1). Segmentation was run in batch mode with GPU acceleration (NVIDIA CUDA 12.3), and all masks were exported in NIfTI format for subsequent feature extraction.

### 2.6 Feature extraction

Radiomics features were extracted from the T2-weighted images using PyRadiomics (version 3.0.1) in accordance with the Image Biomarker Standardization Initiative (IBSI) guidelines (39). Features were computed from 18 image types: (1) original images; (2) LoG-filtered images (*σ* = 3.0, 4.0, and 5.0 mm); (3) wavelet decompositions (8 combinations, LLL to HHH); (4) square; (5) square-root; (6) logarithm; (7) exponential; (8) gradient; and (9) local binary pattern (LBP) 3D images.

These features spanned six categories: shape (*n* = 14), first-order statistics (*n* = 18), gray-level co-occurrence matrix (GLCM; *n* = 24), gray-level run-length matrix (GLRLM; *n* = 16), gray-level size zone matrix (GLSZM; *n* = 16), and gray-level dependence matrix (GLDM; *n* = 14). In total, 1,774 features were computed per ROI. The complete PyRadiomics configuration file is available in the GitHub repository.

### 2.7 Feature selection

Features were selected through a multi-step strategy. First, computational reproducibility was assessed using the intraclass correlation coefficient (ICC): a randomly selected subset of 40 patients (120 discs) was processed twice through the complete automated pipeline, and ICC values were computed under a two-way random-effects model for absolute agreement [ICC(2,1)] using the pingouin package (version 0.5.5) in Python. Only features with an ICC above 0.75 were retained (40). Robustness to segmentation-boundary variation was separately assessed by re-extracting features after one-voxel morphological erosion and dilation of the disc masks (*n* = 630 discs) and computing ICC(1,2) across the three conditions (original, eroded, dilated) (8, 41). Second, the dataset was split at the patient level into training (168 patients, 504 discs) and independent test (42 patients, 126 discs) subsets using stratified random sampling (random seed, 4,321), ensuring that all discs from a given patient were assigned to the same subset. All subsequent selection steps were performed exclusively on the training set. Third, the Kruskal–Wallis *H* test with Bonferroni correction was applied, retaining features with an adjusted *p* < 0.05. Fourth, near-zero-variance features were removed using the nearZeroVar function of the caret package (version 7.0–1) in R. Finally, for each feature pair with a Spearman correlation coefficient above 0.9, the feature with weaker discriminative ability was excluded. After these steps, 517 features (29.1% of the original 1,774) were retained for model development.

### 2.8 Model development and internal validation

Ten machine learning algorithms representing five modeling paradigms were evaluated: (1) tree-based methods (Decision Tree, Random Forest, XGBoost, and CatBoost); (2) linear models (Lasso, Ridge, and multinomial logistic regression); (3) an instance-based method (K-Nearest Neighbors); (4) a probabilistic model (Naive Bayes); and (5) deep learning (Neural Network). Hyperparameters were optimized on the training set via grid search with patient-grouped 5-fold cross-validation (Supplementary Table S2).

Class imbalance was addressed by assigning sample weights inversely proportional to class frequency for the eight algorithms that support this option; K-Nearest Neighbors and Naive Bayes were trained with default settings.

Model performance was evaluated using macro-averaged metrics to give each class equal weight: AUC, accuracy, sensitivity, specificity, precision, and F1-score. Multi-class AUC was calculated by the Hand and Till method (42). Bias-corrected and accelerated (BCa) bootstrap with 1,000 resamples was used to estimate 95% confidence intervals (CIs), and pairwise comparisons were performed by bootstrap resampling (1,000 iterations) with Bonferroni correction for multiple comparisons.

CatBoost was pre-specified as the primary model. The remaining nine algorithms were trained on the same 517 features and served solely as comparators. For the primary model, grade-specific receiver operating characteristic (ROC) curves and a multi-class confusion matrix were constructed. All analyses were performed with the caret (version 7.0–1), catboost (version 1.2.8), and pROC (version 1.18.5) packages in R.

### 2.9 Comparison of alternative approaches

To assess whether three-dimensional radiomics contributed information beyond the mid-sagittal plane used for Pfirrmann grading and simpler descriptors, five comparators were evaluated: mid-sagittal two-dimensional radiomics, five scalar measurements, a frozen ImageNet embedding, a frozen RadImageNet embedding, and disc volume. All comparators used the same patient-level split and CatBoost specification.

Two-dimensional features were extracted without through-plane interpolation (9) from the TotalSpineSeg disc-mask slice with the largest area along the shortest (left–right) axis, using PyRadiomics in force2D mode (in-plane spacing, 1 × 1 mm). Feature reduction followed the same procedure as the three-dimensional analysis.

Five scalars from that slice were modeled separately: disc-height index (mean anterior, middle, and posterior disc height divided by the adjacent vertebral-body interval) (11); the normalized peak signal-intensity difference, (*H*_disc_−*H*_CSF_)/|*H*_CSF_|, where *H* is the histogram mode and CSF denotes cerebrospinal fluid (7); the disc-to-CSF mean signal ratio; mid-sagittal disc area; and 2D sphericity (4πA/P^2^). CSF was defined as voxels at or above the 75th percentile in a posterior strip spanning the disc height.

The CNN embeddings used the same slice. The disc-mask bounding box was expanded by 8 voxels, resized to 224 × 224, and encoded with a frozen ImageNet-pretrained ResNet50 (IMAGENET1K_V2; 2,048-dimensional output after removal of the classification head) (43, 44). The same encoding procedure was repeated using the publicly available RadImageNet ResNet50 weights (45), a radiology-pretrained backbone applied with that checkpoint's native input scaling; neither encoder was fine-tuned. Horizontal flipping was not applied. Each embedding was reduced to 256 dimensions on the training set.

To determine whether grade discrimination was accounted for by disc size alone (8), three-dimensional disc volume was modeled as a single predictor. Volume was defined as the PyRadiomics MeshVolume (mm^3^) computed from the three-dimensional disc mask, not a mid-sagittal measurement. Spearman correlations between each of the 517 retained features and MeshVolume were also computed. As a sensitivity analysis, each of the 517 features was linearly residualized on MeshVolume using coefficients estimated from training discs only, and CatBoost was retrained on the resulting residuals; this residual-adjusted model served as a supplementary analysis and was not substituted for the pre-specified primary model.

### 2.10 External validation

To evaluate the transportability of the primary model beyond the single-center acquisition, the 517-feature CatBoost classifier was applied without feature re-selection to discs at L3–S1 on conventional sagittal T2-weighted images in the public multi-vendor SPIDER dataset (46; Zenodo 10159290). The released expert reference masks were used in place of TotalSpineSeg. SPACE series and cases with a non-standard number of lumbar vertebrae were excluded. Features were extracted using the same three-dimensional settings as in the internal analysis. No hyperparameter search was performed on SPIDER.

### 2.11 Model interpretability analysis

To enhance the interpretability of the primary model, SHAP analysis was performed (47), with SHAP values computed via TreeSHAP. Global feature importance was determined by ranking features by their mean absolute SHAP value across all samples. Grade-specific importance was also assessed, with SHAP values computed separately for each Pfirrmann grade (I–V). In addition, grade-wise association tests, comparison against simple measurements, and ablation of a reduced feature subset were performed. All analyses were conducted using the catboost package (version 1.2.10) and the SciPy package (version 1.18.0) in Python.

### 2.12 Statistical analysis

Descriptive statistics were used to summarize baseline clinical characteristics. Mean ± standard deviation was used for continuous variables, while categorical variables were expressed as frequencies. Statistical methods for feature selection, model validation, and interpretability analysis have been described in the corresponding sections above. Statistical significance was defined as a two-sided *p* value below 0.05. R (version 4.4.2) and Python (version 3.12.0) served as the primary platforms for data analysis.

## 3 Results

### 3.1 Patient characteristics

A total of 210 patients were enrolled, and three lumbar discs (L3–L4, L4–L5, and L5–S1) were assessed in each, yielding 630 discs for analysis. The baseline clinical characteristics are summarized in Table 1.

**Table 1:** Baseline clinical characteristics of participants (*n* = 210).

Characteristic	Value
Age (years, mean ± SD)	47.7 ± 16.9
Age range (years)	21–74
Sex (male/female, n)	82/128
BMI (kg/m^2^, mean ± SD)	24.6 ± 4.9
Symptom duration (months, mean ± SD)	15.0 ± 3.0
VAS score (mean ± SD)	5.5 ± 2.6

BMI, body mass index; VAS, visual analogue scale; SD, standard deviation.

Each disc was graded independently by two radiologists. Disagreement occurred in 258 of 630 discs (41.0%), with no discrepancy exceeding one grade, and the weighted *κ* was 0.804 (95% CI: 0.777–0.831). Disagreements clustered at the Grade III/IV boundary (173 cases, 67.1% of all disagreements), followed by the Grade II/III boundary (63 cases, 24.4%), reflecting the inherent difficulty of distinguishing mid-range degenerative changes even for experienced radiologists. This inter-observer variability served as a clinical benchmark for model performance (Supplementary Table S3). After adjudication, the final distribution of Pfirrmann grades was imbalanced: Grade IV was the most prevalent (36.2%), whereas Grades I and V were minority classes (4.8% and 4.3%, respectively; Table 2).

**Table 2:** Distribution of Pfirrmann grades.

Pfirrmann Grade	*n*	%
I (Normal)	30	4.8
II (Mild degeneration)	178	28.3
III (Moderate degeneration)	167	26.5
IV (Severe degeneration)	228	36.2
V (Advanced degeneration)	27	4.3
**Total**	**630**	**100**.**0**

### 3.2 Automated segmentation performance

Using TotalSpineSeg, all 630 intervertebral discs from the 210 patients were automatically segmented in 24 min, with a mean processing time of 6.9 s per case. Representative results are shown in Figure 2, demonstrating accurate delineation of the discs at the L3–L4, L4–L5, and L5–S1 levels on sagittal T2-weighted MRI.

**Figure 2:**
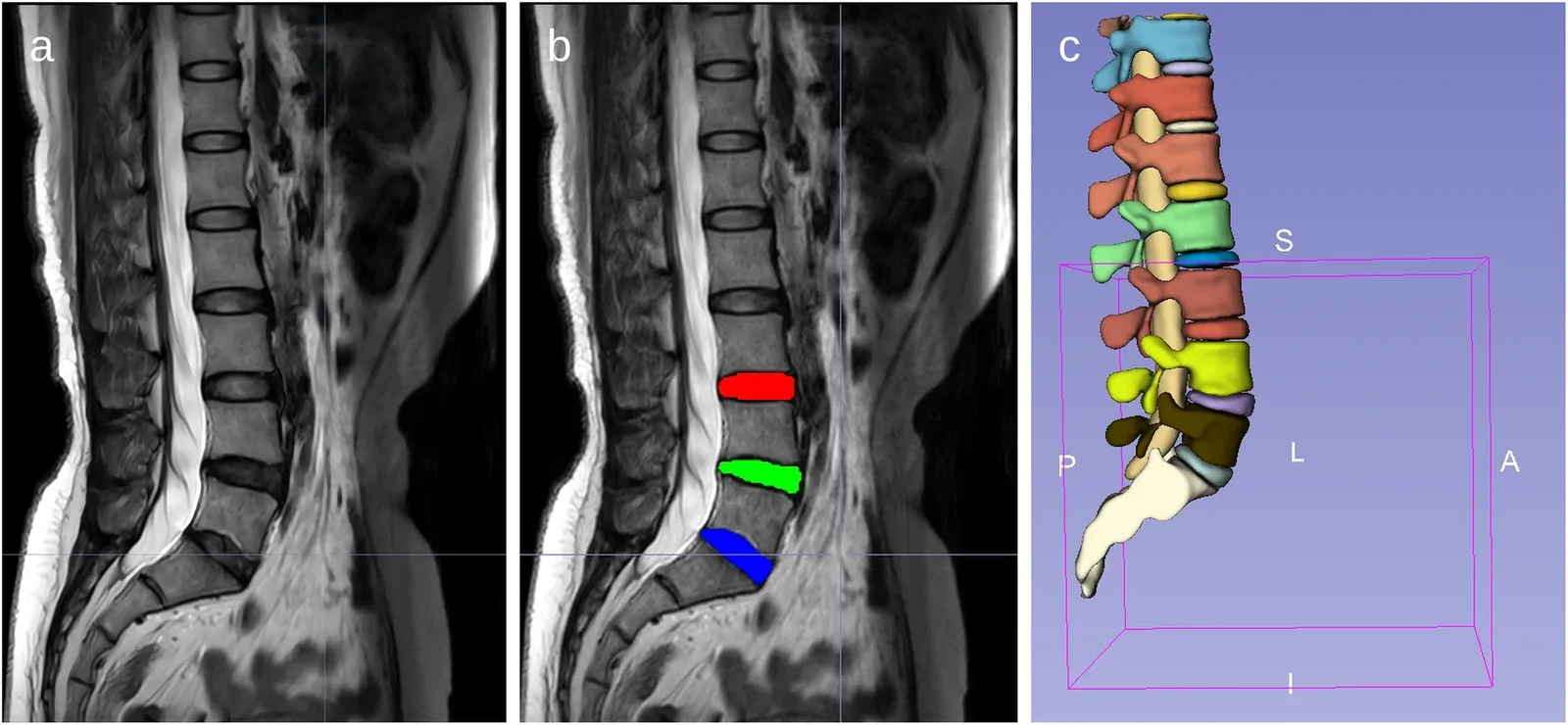
Representative example of automated intervertebral disc segmentation using TotalSpineSeg. **(a)** Original sagittal T2-weighted MRI of the lumbar spine; **(b)** segmentation mask overlay demonstrating the three target intervertebral discs: L3–L4 (red), L4–L5 (green), and L5–S1 (blue); **(c)** three-dimensional volume rendering of the complete segmentation masks visualized in 3D Slicer (version 5.6.1). Vertebral body masks in **(c)** were used to compute disc-height index (adjacent vertebral heights); disc masks were used for radiomics and the remaining simple features. S, superior; I, inferior; A, anterior; P, posterior; L, left.

Of the 1,774 features initially extracted, 1,762 (99.3%) demonstrated an ICC above 0.75; the remaining 12 (0.7%) were excluded. In a separate perturbation analysis, after one-voxel erosion and dilation of the disc masks, 45.3% of re-extracted features showed an ICC(1,2) above 0.75 (median ICC 0.713; *n* = 630 discs). Among the primary-model features with a perturbation ICC, 144 (34.4%) retained an ICC above 0.75 (Supplementary Table S4).

### 3.3 Feature selection

The multi-step selection process progressively reduced the feature set while preserving discriminative features. ICC-based filtering (ICC > 0.75) left 1,762 features. The Kruskal–Wallis *H* test with Bonferroni correction, applied to the training set (*n* = 504), identified 1,548 features with significant discriminative power across Pfirrmann grades (adjusted *p* < 0.05). Subsequent near-zero-variance filtering and correlation-based elimination (|*ρ*| > 0.9) removed a further 1,031 redundant features, yielding a final set of 517 features (29.1% of the original 1,774) for model development. The composition of this set is detailed in Table 3.

**Table 3:** Composition of the selected feature set.

Feature Source	Extracted (*n*)	Retained (*n*)	Retention Rate
Original	102	25	24.5%
LoG (*σ* = 3.0, 4.0, 5.0 mm)	264	86	32.6%
Wavelet (8 decompositions)	704	179	25.4%
Mathematical transforms	352	90	25.6%
Gradient	88	39	44.3%
LBP-3D (3 parameters)	264	98	37.1%
**Total**	**1,774**	**517**	**29**.**1%**

LoG, Laplacian of Gaussian; LBP-3D, local binary pattern 3D; mathematical transforms include square, square root, logarithm, and exponential.

### 3.4 Comparison of machine learning models

The comparative performance of the ten algorithms on the independent test set (*n* = 126) is presented in Figure 3. The pre-specified primary model (CatBoost) achieved a macro-averaged AUC of 0.936 (95% CI: 0.913–0.958). Across all models, sensitivity ranged from 47.0% to 74.9%, and specificity from 89.5% to 93.1%.

**Figure 3:**
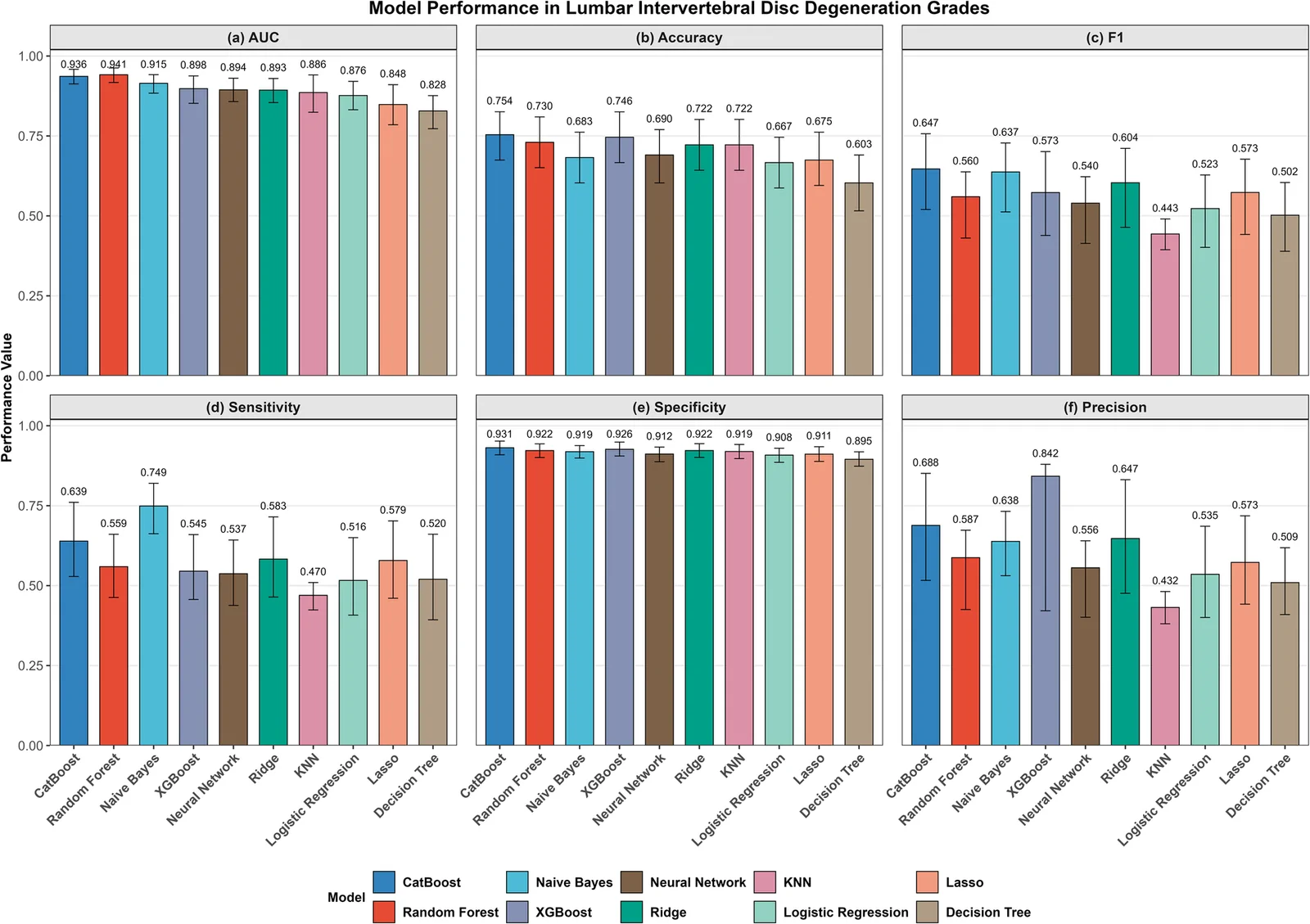
Comparison of ten machine learning algorithms for Pfirrmann grading. Results are from the test set (*n* = 126). Each bar plot shows one of six metrics: **(a)** AUC, **(b)** accuracy, **(c)** F1-score, **(d)** sensitivity, **(e)** specificity, and **(f)** precision. Error bars show 95% confidence intervals (BCa bootstrap, 1,000 resamples). AUC, area under the receiver operating characteristic curve.

Pairwise bootstrap comparisons showed that the CatBoost macro-averaged AUC did not differ from that of Random Forest (*p* = 0.392) but exceeded those of the remaining eight algorithms (all *p* < 0.05). CatBoost was the pre-specified primary model; the remaining algorithms are reported for comparison only.

### 3.5 Performance of the primary model

The classification performance of the primary model (CatBoost) is shown in Figure 4. The one-versus-rest ROC curves revealed that discriminative ability varied across Pfirrmann grades, being highest for Grade V (AUC = 0.978; 95% CI: 0.940–1.000) and lowest for Grade III (AUC = 0.876; 95% CI: 0.806–0.929). The extreme grades (I, II, IV, and V) were more readily distinguished than the intermediate grade (III).

**Figure 4:**
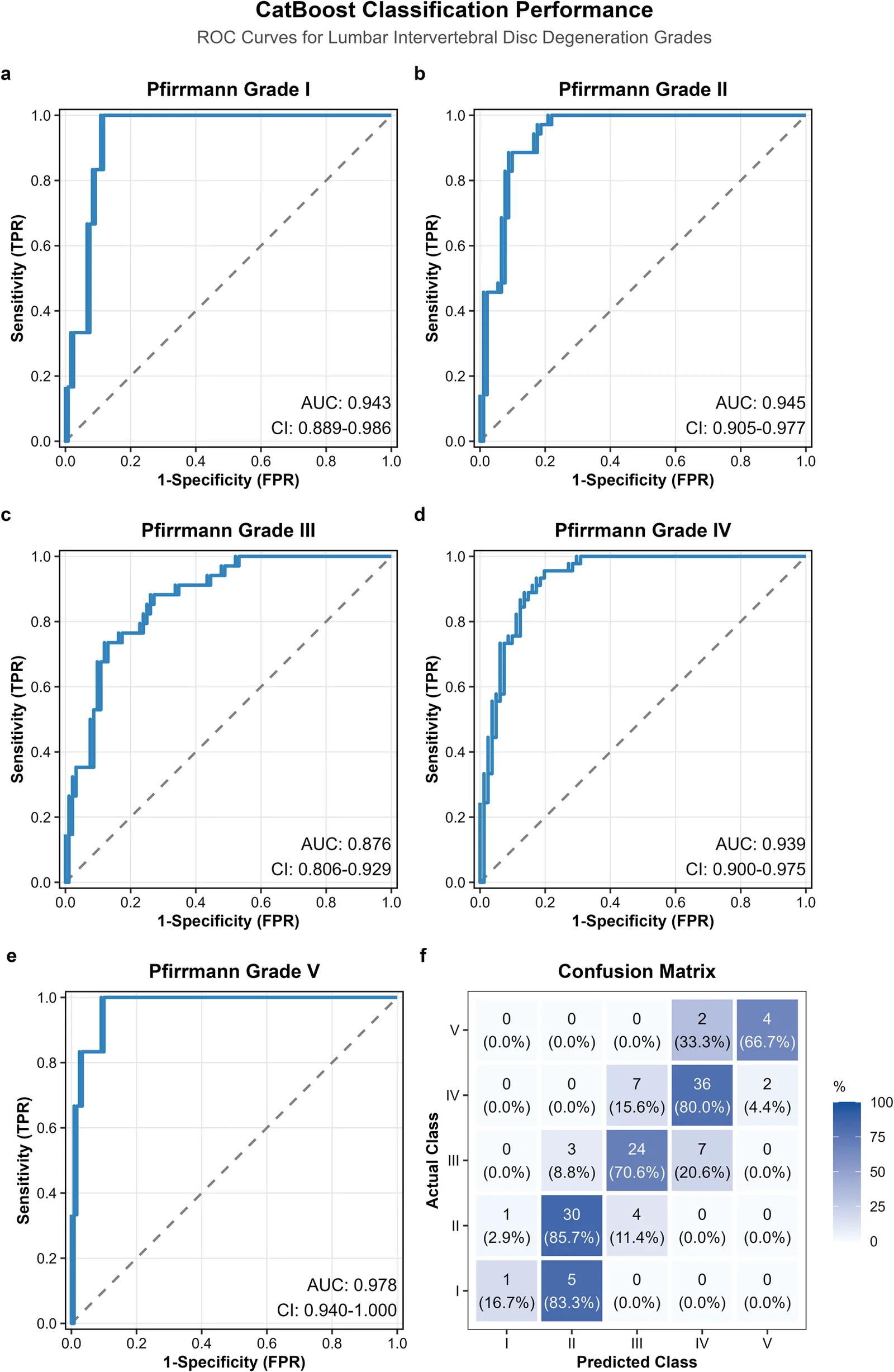
Test set performance of catBoost (*n* = 126). **(a–e)** ROC curves (one-versus-rest) for each Pfirrmann grade **(i–v)**, with AUC and 95% CI. **(f)** Confusion matrix showing predicted versus actual grades. Values are counts, with percentages in parentheses. The diagonal elements indicate correct classifications. ROC, receiver operating characteristic; CI, confidence interval.

The confusion matrix (Figure 4F) showed that all misclassifications occurred between adjacent grades (e.g., Grade I as II, and Grade III as II or IV), with no error spanning two or more grades. Detailed performance metrics for each grade are summarized in Table 4.

**Table 4:** Grade-specific classification performance of the catBoost model.

Pfirrmann Grade	*n*	AUC (95% CI)	Sensitivity (%)	Specificity (%)
I	6	0.943 (0.889–0.986)	16.7	99.2
II	35	0.945 (0.905–0.977)	85.7	91.2
III	34	0.876 (0.806–0.929)	70.6	88.0
IV	45	0.939 (0.900–0.975)	80.0	88.9
V	6	0.978 (0.940–1.000)	66.7	98.3

*n*, number of test-set discs of the corresponding grade; AUC, area under the receiver operating characteristic curve; CI, confidence interval. Estimates for Grades I and V are each based on six discs and are reported descriptively.

### 3.6 Comparison of alternative approaches

On the internal test set, macro-averaged AUC was highest for three-dimensional radiomics (0.936; 95% CI: 0.913–0.958), followed by mid-sagittal two-dimensional radiomics (0.916; 0.884–0.945), the frozen RadImageNet embedding (0.874; 0.834–0.910), the frozen ImageNet embedding (0.864; 0.828–0.899), and the strongest simple measurement (disc-to-CSF mean signal ratio, 0.760; 0.714–0.807; Table 5).

**Table 5:** Internal test-set macro AUC for the primary model and five comparators under the same patient-level split and catBoost specification.

Approach	*n* features	Macro AUC (95% CI)
3D radiomics (primary)	517	0.936 (0.913–0.958)
2D mid-sagittal radiomics	244	0.916 (0.884–0.945)
Frozen RadImageNet ResNet50	256	0.874 (0.834–0.910)
Frozen ImageNet ResNet50	256	0.864 (0.828–0.899)
Disc-to-CSF signal ratio	1	0.760 (0.714–0.807)
Disc volume (MeshVolume)	1	0.552 (0.491–0.622)

AUC, area under the receiver operating characteristic curve; CI, confidence interval.

Paired bootstrap comparison showed that the difference (*Δ*) between three-dimensional and two-dimensional radiomics was not significant (*Δ* = 0.020; *p* = 0.079). Both radiomic models significantly exceeded the frozen RadImageNet embedding (three-dimensional, *p* < 0.001; two-dimensional, *p* = 0.017), the frozen ImageNet embedding (three-dimensional, *p* < 0.001; two-dimensional, *p* = 0.023), and the disc-to-CSF ratio (both *p* < 0.001). The two frozen embeddings did not differ significantly from each other (*Δ* = 0.010; 95% CI: −0.035 to 0.052; *p* = 0.673).

Disc volume alone yielded a macro-averaged AUC of 0.552 (0.491–0.622), lower than all other comparators (all *p* < 0.001). Of the 517 features, 20.3% had |*ρ*| > 0.5 with MeshVolume (median |*ρ*| = 0.234). After residual adjustment, macro-averaged AUC was 0.924 (0.897–0.950), only 0.012 below the pre-specified primary result of 0.936, which remains the reported estimate.

### 3.7 External validation

Applied without feature re-selection to 597 discs from 199 patients in the SPIDER dataset (46), the frozen 517-feature CatBoost classifier achieved a macro-averaged AUC of 0.838 (95% CI: 0.816–0.860), 0.098 below the internal primary estimate. This result should be interpreted as evidence of radiomic feature transportability across acquisition protocols, supplementing rather than replacing the internal primary estimate (Supplementary Table S5).

### 3.8 Model interpretability analysis

SHAP analysis identified the key radiomics features driving model predictions (Figure 5). Global feature importance ranking (Figure 5F) revealed the three leading features spanning intensity, shape, and texture domains: (1) log-sigma-4–0-mm-3D_firstorder_Minimum (mean |SHAP| = 0.065), capturing the lowest voxel intensities after LoG filtering; (2) original_shape_Flatness (mean |SHAP| = 0.061), quantifying the degree of plate-like disc morphology; and (3) log-sigma-3-0-mm-3D_glcm_Imc2 (mean |SHAP| = 0.094), a texture feature reflecting gray-level co-occurrence dependence.

**Figure 5:**
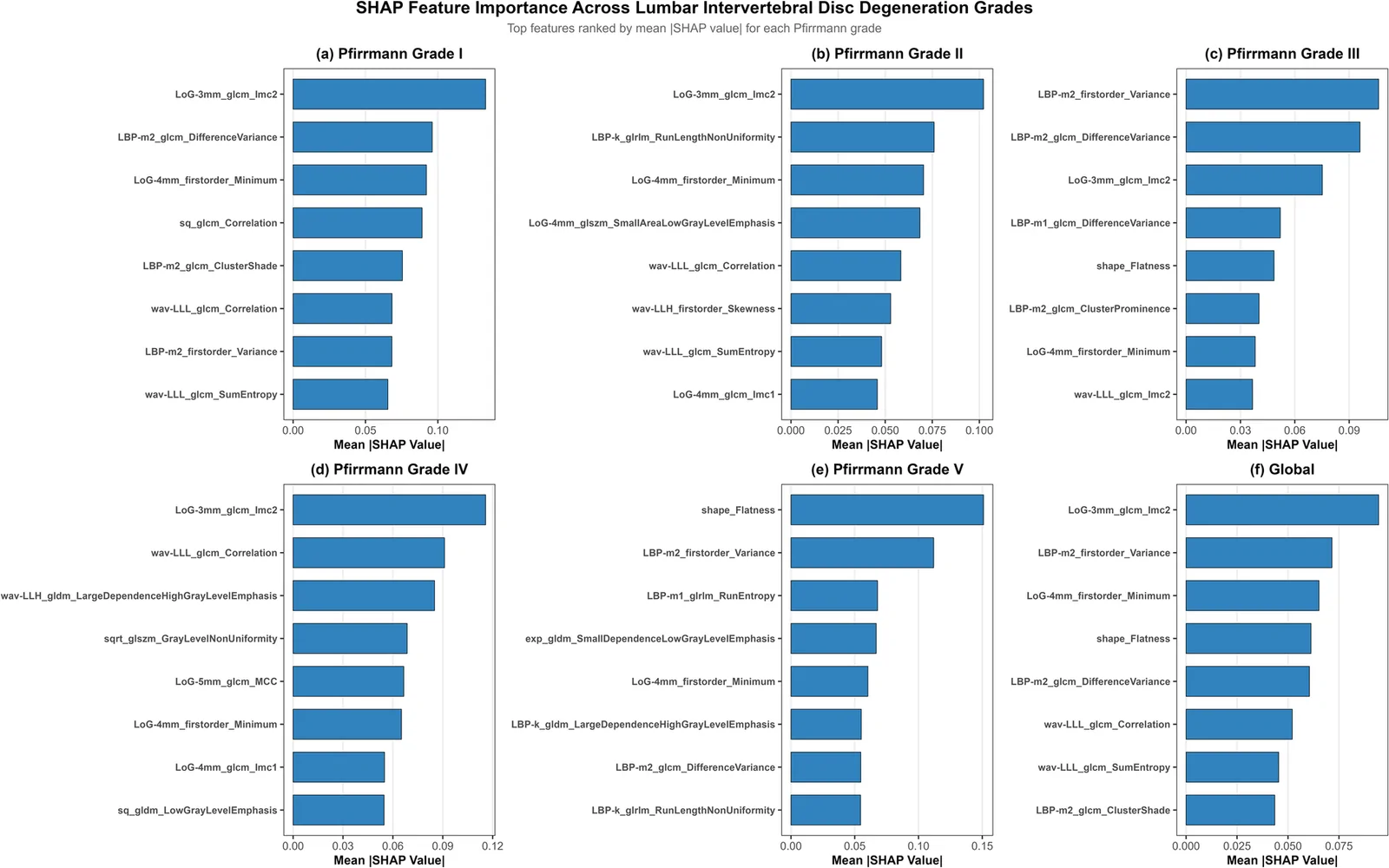
SHAP (SHapley additive exPlanations) feature importance analysis for the catBoost classifier. Bar plots display the top features ranked by mean absolute SHAP value. **(a–e)** Grade-specific importance for Pfirrmann Grades I–V. **(f)** Global importance across all classes. The most influential features include log-sigma-4-0-mm-3D_firstorder_Minimum (intensity), original_shape_Flatness (shape), and log-sigma-3-0-mm-3D_glcm_Imc2 (texture).

Across the 517 features, the median absolute Spearman correlation with Pfirrmann grade was 0.351. Imc2 and the LoG 4 mm minimum correlated strongly with grade (*ρ* = −0.75 and +0.76). The radiomic feature set was largely uncorrelated with disc-height index and peak signal-intensity difference (0.8% and 0% of features with |*ρ*| > 0.5, respectively). A 10-feature subset achieved a macro-averaged AUC of 0.922, compared with 0.936 for the full primary model.

## 4 Discussion

LDD is a major contributor to low back pain, the leading cause of disability worldwide, and therefore requires reliable methods of assessment (1). Visual Pfirrmann grading, however, suffers from inherent inter-observer variability: prior studies report only 66%–83% agreement (4, 5), and our cohort confirmed this limitation (59.0% exact agreement; weighted *κ* = 0.804). These findings are consistent with the literature and underscore the subjectivity of visual grading. Although radiomics offers objective, quantitative evaluation, its clinical adoption has been hindered by labor-intensive, operator-dependent manual segmentation (23). TotalSpineSeg enables fully automated spine segmentation but had not previously been applied to LDD grading. In the present study, TotalSpineSeg processed 210 cases (630 discs) in 24 min (6.9 s per case), substantially reducing processing time and eliminating operator-dependent contouring from the radiomics workflow (23, 48).

Among the ten algorithms evaluated, the CatBoost model achieved a macro-averaged AUC of 0.936 (95% CI: 0.913–0.958). Pairwise comparison showed that this estimate did not differ from that of Random Forest (*p* = 0.392) but exceeded those of the remaining eight algorithms (all *p* < 0.05). The performance of our model is comparable to that reported in previous LDD classification studies: Liawrungrueang et al. reported a test AUC of 0.92 for Pfirrmann grading with a CNN-YOLO model on T2-weighted MRI (12), and a recent multi-center study by Bai et al. achieved an external-validation AUC of approximately 0.90 using ensemble voting with radiomics from wavelet-fused MRI (20). The superior performance of CatBoost can be attributed to its technical characteristics: ordered boosting and symmetric tree structures inherently mitigate overfitting, while built-in class weighting effectively handles imbalanced distributions. These properties are particularly advantageous for multi-class classification with limited sample sizes (49, 50). Compared with linear models such as logistic regression, non-linear ensemble algorithms like CatBoost better handle high-dimensional feature spaces and capture the complex non-linear patterns in imaging data (51). Compared with end-to-end deep learning, handcrafted radiomics combined with conventional machine learning can achieve comparable performance with limited samples while offering greater training stability and interpretability (52–55).

The grade-specific analysis offered insight into the classification challenges across the degeneration spectrum. Grades I and II showed strong discriminative ability (AUC = 0.943 and 0.945, respectively), because their bright, homogeneous signal and preserved nucleus-annulus distinction yield radiomic signatures clearly distinct from those of advanced degeneration (4). Similarly, Grade V achieved the highest discriminative performance (AUC = 0.978), reflecting the unmistakable imaging features of end-stage degeneration: hypointense signal with complete loss of disc height (4, 56).

In contrast, the intermediate grades showed lower classification performance. Grade III was the most difficult to classify, with the lowest AUC (0.876). This parallels the inter-observer analysis, in which Grade III also had the lowest exact agreement among radiologists. This convergence of human and machine difficulty suggests that the challenge is intrinsic to transitional imaging features (such as intermediate gray signal and indistinct nucleus-annulus differentiation) rather than a limitation specific to either approach (4–6). The confusion matrix showed that all misclassified Grade III cases were assigned to adjacent grades, indicating that the model captures the underlying biological continuum rather than producing random errors. Grade IV achieved an AUC of 0.939, as its dark gray signal and moderate height reduction share features with both neighboring grades (4). The confinement of misclassifications to adjacent grades confirms that model performance is ultimately bounded by the continuous nature of disc degeneration.

Three-dimensional radiomics yielded the highest discriminative performance within the study cohort and was retained as the pre-specified primary analysis, with a modest advantage over mid-sagittal two-dimensional radiomics. On the same patient-level split, macro-averaged AUC was 0.936 (95% CI: 0.913–0.958) for three-dimensional features and 0.916 (0.884–0.945) for two-dimensional features (Table 5); the 0.020 difference was not significant (*p* = 0.079). Because automated three-dimensional masks inherently locate the grading plane for the two-dimensional arm (8), both representations benefit from the same segmentation. The modest incremental gain from volumetric analysis is consistent with Pfirrmann grade being assigned on the mid-sagittal image and with a slice spacing of 4.0 mm (9).

Beyond the automation and interpretability discussed above, the radiomic models discriminated Pfirrmann grade more accurately than simpler alternatives. Both radiomic models exceeded frozen ResNet50 embeddings of the mid-sagittal plane (RadImageNet, 0.874, 0.834–0.910; ImageNet, 0.864, 0.828–0.899) as well as the strongest simple measurement, the disc-to-CSF mean signal ratio (0.760; 0.714–0.807). Of note, these embeddings are frozen encoders rather than networks trained end to end on Pfirrmann labels (12–16). Disc-height index and peak signal-intensity difference, widely used as interpretable alternatives (7, 11), performed below the disc-to-CSF ratio. Disc MeshVolume alone yielded a macro-averaged AUC of only 0.552 (0.491–0.622), and only 20.3% of the 517 retained features had |*ρ*| > 0.5 with volume, confirming that model discrimination is not driven by disc size (8, 57).

External validation demonstrated adequate generalizability. Applied without feature re-selection to 597 L3–S1 discs from 199 patients in the multi-vendor SPIDER dataset (46), the frozen 517-feature classifier achieved a macro-averaged AUC of 0.838 (0.816–0.860), only 0.098 below the internal primary estimate of 0.936, despite the transition from a single-center 3.0 T protocol to conventional sagittal T2-weighted images acquired on scanners from two manufacturers. Performance was comparable between Philips and Siemens examinations (0.821 and 0.820). These findings suggest that the radiomic signature is transportable across vendors and acquisition protocols; however, prospective testing of the full automated pipeline at additional centers remains a necessary next step.

SHAP analysis quantifies the contribution of each feature and enables global importance ranking, thereby improving model transparency and aiding the identification of candidate imaging biomarkers (47, 58–60). Our analysis identified three features with clear pathophysiological significance. First, log-sigma-4-0-mm-3D_firstorder_Minimum represented intensity. This first-order statistic represents the lowest signal intensity after LoG filtering; by capturing the darkest voxel intensities, it reflects the T2 signal loss associated with proteoglycan degradation and water depletion during degeneration (3, 61, 62). Second, original_shape_Flatness quantifies the degree of plate-like disc morphology, with lower values indicating a more flattened geometry characteristic of Grade V. As the nucleus pulposus loses hydration and the annulus fibrosus weakens, the disc deviates from its normal elliptical geometry, making this feature a marker of macroscopic structural change (2). Third, log-sigma-3-0-mm-3D_glcm_Imc2, a texture feature derived from LoG filtering, is an informational measure of gray-level co-occurrence dependence. Lower values indicate reduced dependence between neighboring gray levels, consistent with the loss of organized internal architecture as the disc becomes more uniformly hypointense (63, 64). Collectively, these three features correspond to established pathophysiology: signal-intensity loss reflects biochemical degradation, decreased flatness indicates morphological deterioration, and altered texture reflects structural disorganization (2, 62, 65). This concordance between data-driven feature importance and domain knowledge supports the biological plausibility of our model.

This study has several limitations. First, the development cohort is retrospective and single-center. The SPIDER analysis used expert reference masks rather than TotalSpineSeg and therefore assessed radiomic-feature transportability, not the full automated pipeline; prospective multi-center evaluation of the complete pipeline is still needed (25). Second, although the sample of 630 discs is comparable to that of similar radiomics studies (66, 67), class imbalance with underrepresentation of the extreme grades (I and V) may affect model calibration; future studies should expand training datasets and achieve more balanced grade distributions through multi-institutional data pooling (68). Third, this study focused on imaging-based Pfirrmann grading without correlation to clinical outcomes; further research is warranted to examine the relationships between radiomics-predicted grades and pain severity, functional disability, and treatment response (69). Fourth, the original feature-wise ICC was obtained by repeating the identical automated pipeline and therefore reflects computational reproducibility rather than scan–rescan or inter-observer stability (24, 41). Repeat scanning and dual manual contouring were not performed; both would provide a direct test of the expected advantage of automated segmentation for feature reproducibility (22, 23).

## 5 Conclusion

This study developed an automated radiomics pipeline integrating TotalSpineSeg for Pfirrmann grading of LDD. Three-dimensional radiomic features contributed the greatest discriminative information among the evaluated approaches, yielding a modest increment over mid-sagittal two-dimensional radiomics and significantly outperforming simple measurements and frozen ResNet50 embeddings. The approach addresses the segmentation bottleneck in radiomics research by enabling rapid, operator-independent processing. Among the ten machine learning algorithms assessed, the CatBoost model achieved strong discriminative performance. SHAP-based interpretability analysis identified intensity, shape, and texture features as candidate imaging biomarkers with pathophysiological relevance to disc degeneration. The pipeline provides an objective, interpretable solution for standardized LDD assessment, with the potential to reduce diagnostic workload and inter-observer variability in clinical practice. Further external validation of the full automated pipeline across additional imaging protocols and patient populations is warranted.

## Data Availability

Publicly available datasets were analyzed in this study. This data can be found here: The extracted radiomic feature data and the code used in this study, including the complete PyRadiomics configuration file (extraction_params.yaml) and the radiomic feature data (radiomic_feature_data.xlsx), are openly available in the GitHub repository "Radiomics-TotalSpineSeg-LDD" at https://github.com/Cookie-Guo/Radiomics-TotalSpineSeg-LDD. The external validation used the publicly available SPIDER dataset, which can be found in the Zenodo repository at https://doi.org/10.5281/zenodo.10159290 (record 10159290). The original MRI data are not publicly available due to ethical restrictions and patient privacy concerns, but are available from the corresponding authors upon reasonable request and with the approval of the Ethics Committee of Wangjing Hospital, China Academy of Chinese Medical Sciences.
