# Supplementary material for: Automated radiomics pipeline integrating TotalSpineSeg for Pfirrmann grading of lumbar disc degeneration with SHAP-based interpretability
Source: Front Med (Lausanne). 2026 Sep 29;13:1929252. doi: 10.3389/fmed.2026.1929252 (PMC13658075; doi:10.3389/fmed.2026.1929252)
Supplement: Supplementary file 1 [file Table1.docx]

Supplementary Material

# Supplementary Tables

This file contains the Supplementary Tables cited in the main text: Table S1 (CLEAR checklist), Table S2 (hyperparameter search space and selected values), Table S3 (inter-observer agreement analysis), Table S4 (feature robustness to one-voxel mask perturbation), and Table S5 (external evaluation in the SPIDER cohort).

Supplementary Table S1. CLEAR Checklist v1.0.

Note: Use the checklist in conjunction with the main text for clarification of all items. Yes, details provided; No, details not provided; n/e, not essential; n/a, not applicable; Page, page number or location.

| **Section** | **No.** | **Item** | **Yes** | **No** | **n/a** | **Page** |
| --- | --- | --- | --- | --- | --- | --- |
| *Title* | 1 | Relevant title, specifying the radiomic methodology | ✓ |  |  | 1 |
| *Abstract* | 2 | Structured summary with relevant information | ✓ |  |  | 1, 2 |
| *Keywords* | 3 | Relevant keywords for radiomics | ✓ |  |  | 1 |
| *Introduction* | 4 | Scientific or clinical background | ✓ |  |  | 2, 3 |
|  | 5 | Rationale for using a radiomic approach | ✓ |  |  | 2, 3 |
|  | 6 | Study objective(s) | ✓ |  |  | 3 |
| **Method** | | | | | | |
| *Study design* | 7 | Adherence to guidelines or checklists (e.g., CLEAR checklist) | ✓ |  |  | 3 |
|  | 8 | Ethical details (e.g., approval, consent, data protection) | ✓ |  |  | 3, 13 |
|  | 9 | Sample size calculation | ✓ |  |  | 4 |
|  | 10 | Study nature (e.g., retrospective, prospective) | ✓ |  |  | 3 |
|  | 11 | Eligibility criteria | ✓ |  |  | 4 |
|  | 12 | Flowchart for technical pipeline | ✓ |  |  | 3, 21 |
| *Data* | 13 | Data source (e.g., private, public) | ✓ |  |  | 3, 4, 7 |
|  | 14 | Data overlap |  |  | ✓ | n/a |
|  | 15 | Data split methodology | ✓ |  |  | 5 |
|  | 16 | Imaging protocol (i.e., image acquisition and processing) | ✓ |  |  | 4, 5 |
|  | 17 | Definition of non-radiomic predictor variables | ✓ |  |  | 4, 18 |
|  | 18 | Definition of the reference standard (i.e., outcome variable) | ✓ |  |  | 4 |
| *Segmentation* | 19 | Segmentation strategy | ✓ |  |  | 5 |
|  | 20 | Details of operators performing segmentation | ✓ |  |  | 5 |
| *Pre-processing* | 21 | Image pre-processing details | ✓ |  |  | 4, 5 |
|  | 22 | Resampling method and its parameters | ✓ |  |  | 4, 5 |
|  | 23 | Discretization method and its parameters | ✓ |  |  | 5 |
| *Feature extraction* | 24 | Image types (e.g., original, filtered, transformed) | ✓ |  |  | 5 |
|  | 25 | Feature extraction method | ✓ |  |  | 5 |
|  | 26 | Feature classes | ✓ |  |  | 5 |
|  | 27 | Number of features | ✓ |  |  | 5 |
|  | 28 | Default configuration statement for remaining parameters | ✓ |  |  | 5, 13 |
| *Data preparation* | 29 | Handling of missing data |  |  | ✓ | n/a |
|  | 30 | Details of class imbalance | ✓ |  |  | 6 |
|  | 31 | Details of segmentation reliability analysis | ✓ |  |  | 5, 8 |
|  | 32 | Feature scaling details (e.g., normalization, standardization) | ✓ |  |  | 4, 5 |
|  | 33 | Dimension reduction details | ✓ |  |  | 5 |
| *Modeling* | 34 | Algorithm details | ✓ |  |  | 6 |
|  | 35 | Training and tuning details | ✓ |  |  | 6 |
|  | 36 | Handling of confounders | ✓ |  |  | 7, 9 |
|  | 37 | Model selection strategy | ✓ |  |  | 6 |
| *Evaluation* | 38 | Testing technique (e.g., internal, external) | ✓ |  |  | 6, 7 |
|  | 39 | Performance metrics and rationale for choosing | ✓ |  |  | 6 |
|  | 40 | Uncertainty evaluation and measures (e.g., confidence intervals) | ✓ |  |  | 6, 8 |
|  | 41 | Statistical performance comparison (e.g., DeLong's test) | ✓ |  |  | 6, 8, 9 |
|  | 42 | Comparison with non-radiomic and combined methods | ✓ |  |  | 6, 7 |
|  | 43 | Interpretability and explainability methods | ✓ |  |  | 7, 9, 10 |
| *Results* | 44 | Baseline demographic and clinical characteristics | ✓ |  |  | 7, 18 |
|  | 45 | Flowchart for eligibility criteria |  |  | ✓ | n/a |
|  | 46 | Feature statistics (e.g., reproducibility, feature selection) | ✓ |  |  | 8, 19 |
|  | 47 | Model performance evaluation | ✓ |  |  | 8, 9, 19 |
|  | 48 | Comparison with non-radiomic and combined approaches | ✓ |  |  | 9, 19, 20 |
| *Discussion* | 49 | Overview of important findings | ✓ |  |  | 10, 11 |
|  | 50 | Previous works with differences from the current study | ✓ |  |  | 10 |
|  | 51 | Practical implications | ✓ |  |  | 10, 12 |
|  | 52 | Strengths and limitations (e.g., bias and generalizability issues) | ✓ |  |  | 11, 12 |
| **Open Science** | | | | | | |
| *Data availability* | 53 | Sharing images along with segmentation data [n/e] |  |  | ✓ | n/a |
|  | 54 | Sharing radiomic feature data | ✓ |  |  | 13 |
| *Code availability* | 55 | Sharing pre-processing scripts or settings | ✓ |  |  | 13 |
|  | 56 | Sharing source code for modeling | ✓ |  |  | 13 |
| *Model availability* | 57 | Sharing final model files | ✓ |  |  | 13 |
|  | 58 | Sharing a ready-to-use system [n/e] |  |  | ✓ | n/a |

Summary: Yes: 53 items; No: 0 items; n/a: 5 items.

Reference: Kocak B, Baessler B, Bakas S, Cuocolo R, Fedorov A, Maier-Hein L, Mercaldo N, Müller H, Orlhac F, Pinto Dos Santos D, Stanzione A, Ugga L, Zwanenburg A. CheckList for EvaluAtion of Radiomics research (CLEAR): a step-by-step reporting guideline for authors and reviewers endorsed by ESR and EuSoMII. Insights Imaging 2023;14:75. doi: 10.1186/s13244-023-01415-8.

Supplementary Table S2. Hyperparameter search space and selected values for machine learning algorithms.

| **Algorithm** | **Hyperparameter** | **Search Space** | **Selected value** |
| --- | --- | --- | --- |
| Decision Tree | ccp_alpha | 0, 0.01, 0.02, 0.05, 0.1 | 0.05 |
| Random Forest | max_features | 10, 20, 30, 40 | 10 |
| XGBoost | n_estimators | 150, 200, 300 (paired with depth/eta) | 300 |
|  | max_depth | 2, 3, 4 (paired combinations) | 4 |
|  | learning_rate | 0.05, 0.1 (paired combinations) | 0.05 |
|  | gamma | 0 (fixed) | 0 |
|  | colsample_bytree | 0.8 (fixed) | 0.8 |
|  | min_child_weight | 1 (fixed) | 1 |
|  | subsample | 0.8 (fixed) | 0.8 |
| CatBoost (primary) | depth | Pre-specified | 2 |
|  | learning_rate | Pre-specified | 0.05 |
|  | l2_leaf_reg | Pre-specified | 1 |
|  | iterations | Pre-specified | 223 |
|  | class_weights | Inversely proportional to class frequencies | Applied |
| Lasso | alpha | 1 (fixed) | 1 |
|  | lambda | 8 values, log scale, 10⁻³ to 1 | 1.0 |
| Ridge | alpha | 0 (fixed) | 0 |
|  | lambda | 8 values, log scale, 10⁻³ to 10 | 10.0 |
| Neural Network | size (hidden units) | 5, 10, 20 | 20 |
|  | decay (weight decay) | 0.5, 0.1, 0.01 | 0.01 |
| Logistic Regression | decay (weight decay) | 0, 0.1, 0.5, 1.0 | 1.0 |
| K-Nearest Neighbors | k (neighbors) | 3, 5, 7, 9, 11 | 11 |
| Naive Bayes | var_smoothing | 10⁻⁹, 10⁻⁸, 10⁻⁷, 10⁻⁶ | 10⁻⁶ |

Note: Comparison models were tuned by grid search with 5-fold cross-validation grouped by patient on the training set only. CatBoost was pre-specified (depth 2, learning rate 0.05, L2 = 1, 223 iterations, seed 4321) and was not chosen by test-set AUC. Class weights inversely proportional to class frequencies were used for CatBoost and for the tree and linear models, but not for k-NN or naive Bayes.

Supplementary Table S3. Inter-observer agreement analysis for Pfirrmann grading.

| **Metric** | **Value** |
| --- | --- |
| ***Overall agreement*** | |
| Agreement rate | 59.0% (372/630) |
| Cohen's κ (unweighted) | 0.438 (95% CI: 0.384–0.493) |
| Cohen's κ (weighted) | 0.804 (95% CI: 0.777–0.831) |
| Interpretation (Landis & Koch) | Substantial |
| ***Grade-specific agreement*** | |
| Grade I | 100.0% (30/30) |
| Grade II | 80.3% (143/178) |
| Grade III | 34.7% (58/167) |
| Grade IV | 50.0% (114/228) |
| Grade V | 100.0% (27/27) |

CI, confidence interval. Kappa interpretation according to Landis and Koch criteria: <0.20, slight; 0.21–0.40, fair; 0.41–0.60, moderate; 0.61–0.80, substantial; 0.81–1.00, almost perfect/excellent.

Supplementary Table S4. Robustness of radiomic features to one-voxel morphological perturbation of the disc masks (629 discs; original, eroded and dilated conditions).

| **Feature class** | **n features** | **Median ICC(2,1)** | **ICC > 0.75 (%)** |
| --- | --- | --- | --- |
| First-order | 306 | 0.774 | 54.6 |
| GLRLM | 272 | 0.790 | 53.3 |
| GLDM | 238 | 0.718 | 46.6 |
| GLCM | 408 | 0.678 | 40.7 |
| GLSZM | 272 | 0.667 | 38.6 |
| NGTDM | 85 | 0.669 | 29.4 |
| Shape | 14 | 0.612 | 21.4 |
| **All re-extracted features** | **1,595** | **0.713** | **45.3** |
| Primary-model subset | 419 | 0.617 | 34.4 |

Supplementary Table S5. External evaluation of the frozen 517-feature CatBoost classifier in the SPIDER cohort (597 discs, 199 patients), applied without re-selection or re-tuning.

Panel A. Overall and grade-specific discrimination.

| **Metric or grade** | **Value** |
| --- | --- |
| Macro AUC (95% CI) | 0.838 (0.816–0.860) |
| Accuracy | 0.536 (0.497–0.576) |
| Macro sensitivity | 0.521 (0.479–0.560) |
| Macro specificity | 0.878 (0.868–0.888) |
| Quadratic weighted κ | 0.705 (0.654–0.748) |
| Grade I AUC (n = 52) | 0.832 |
| Grade II AUC (n = 93) | 0.861 |
| Grade III AUC (n = 191) | 0.749 |
| Grade IV AUC (n = 146) | 0.833 |
| Grade V AUC (n = 115) | 0.916 |

Panel B. Stratified performance and feature-subset analyses.

| **Stratum or subset** | **n discs** | **Macro AUC** |
| --- | --- | --- |
| Philips | 315 | 0.821 |
| Siemens | 282 | 0.820 |
| 1.5 T | 309 | 0.857 |
| 3.0 T | 288 | 0.807 |
| First-order only (119 features), external | 597 | 0.832 |
| First-order only, internal test set | 126 | 0.930 |
| Shape only (7 features), external | 597 | 0.699 |
| Shape only, internal test set | 126 | 0.799 |

Expert reference masks released with SPIDER were used; TotalSpineSeg was not applied, because SPIDER annotations formed part of its training data. The internal primary estimate was 0.936 (0.913–0.958).
